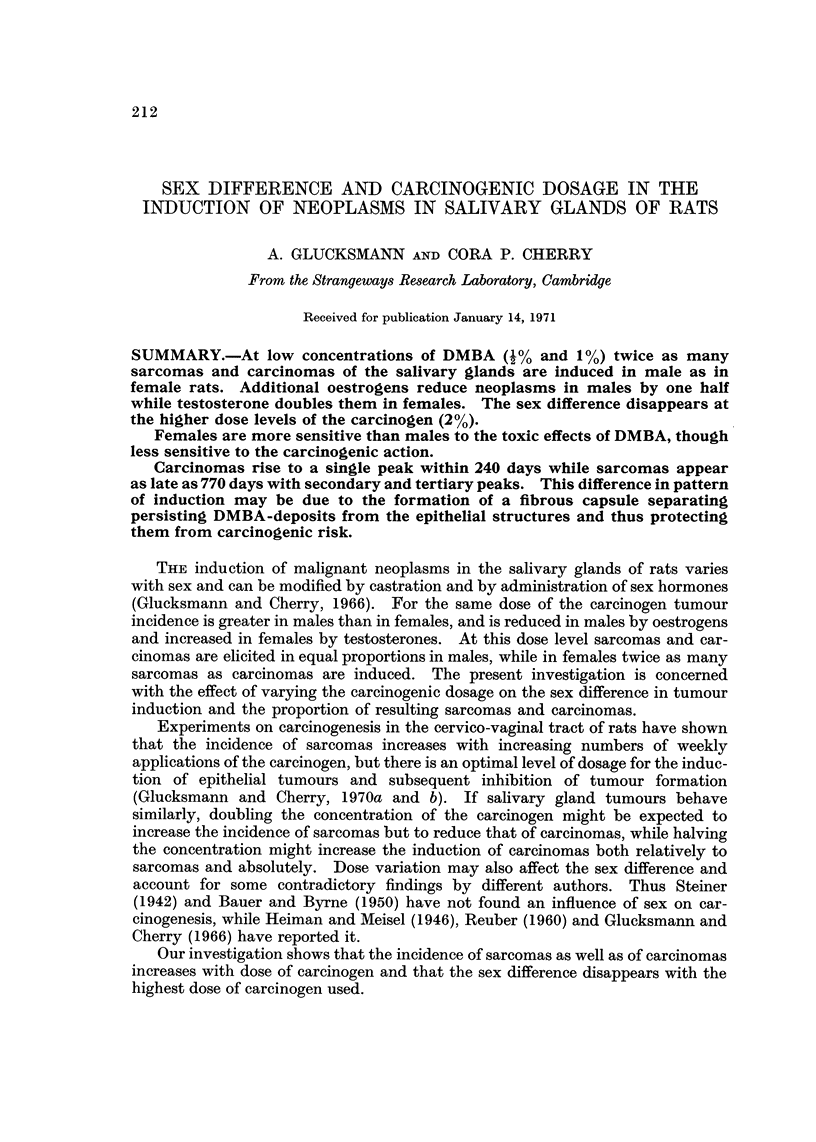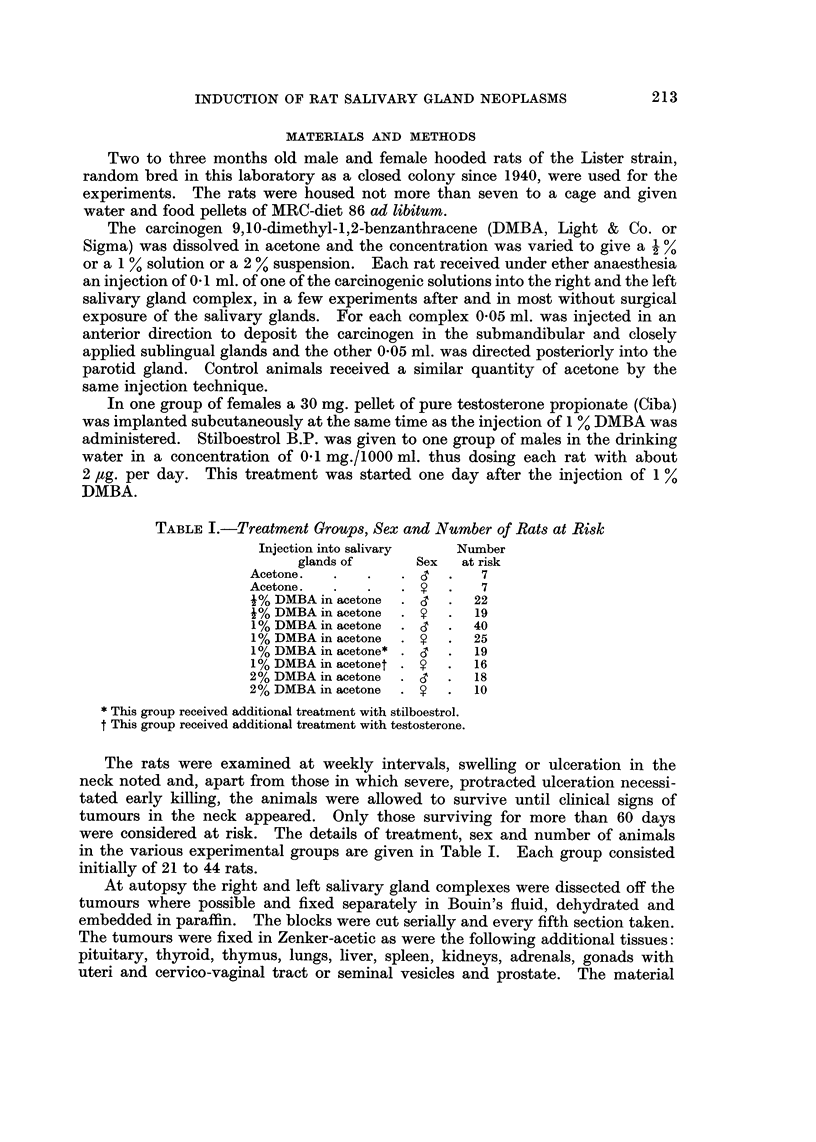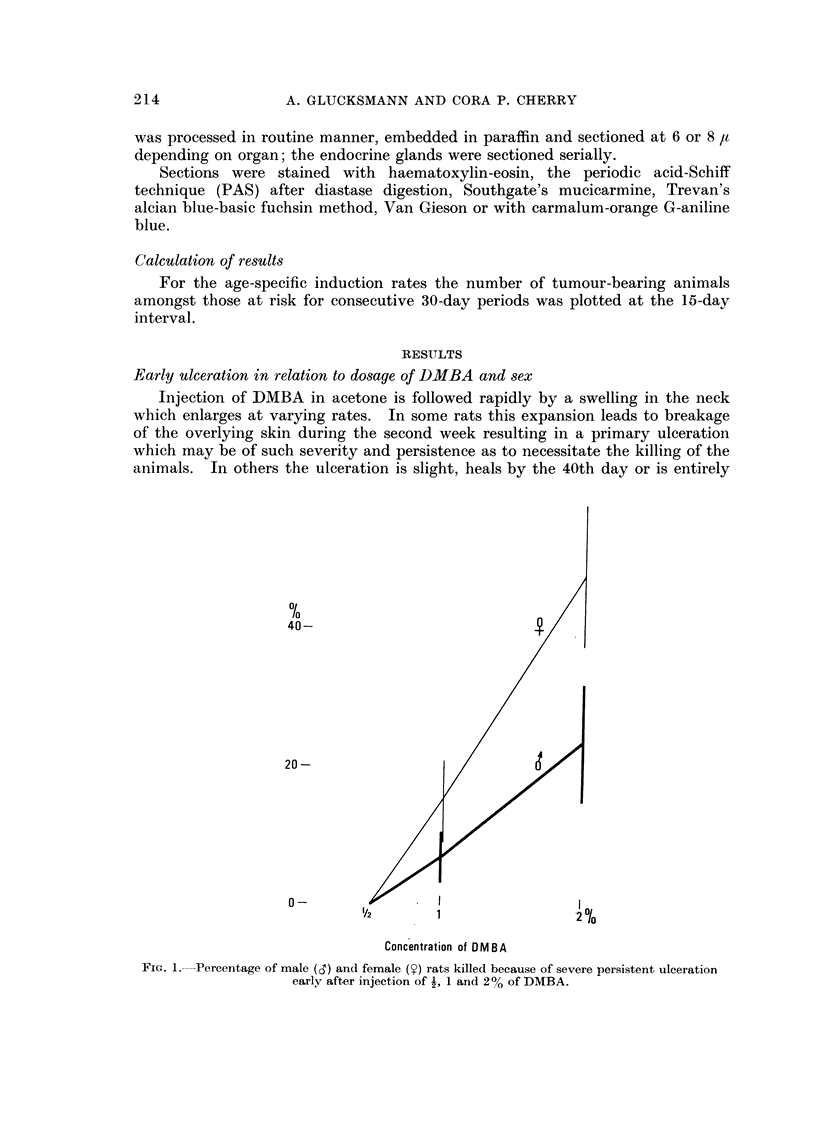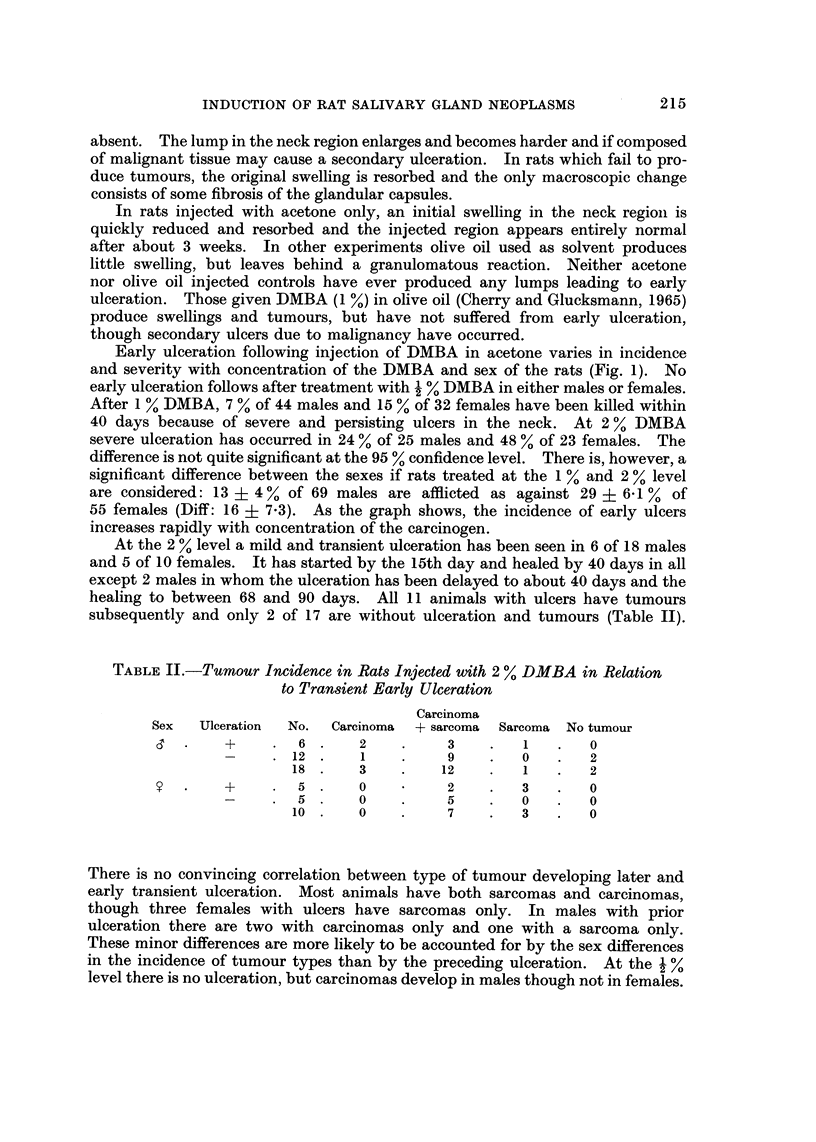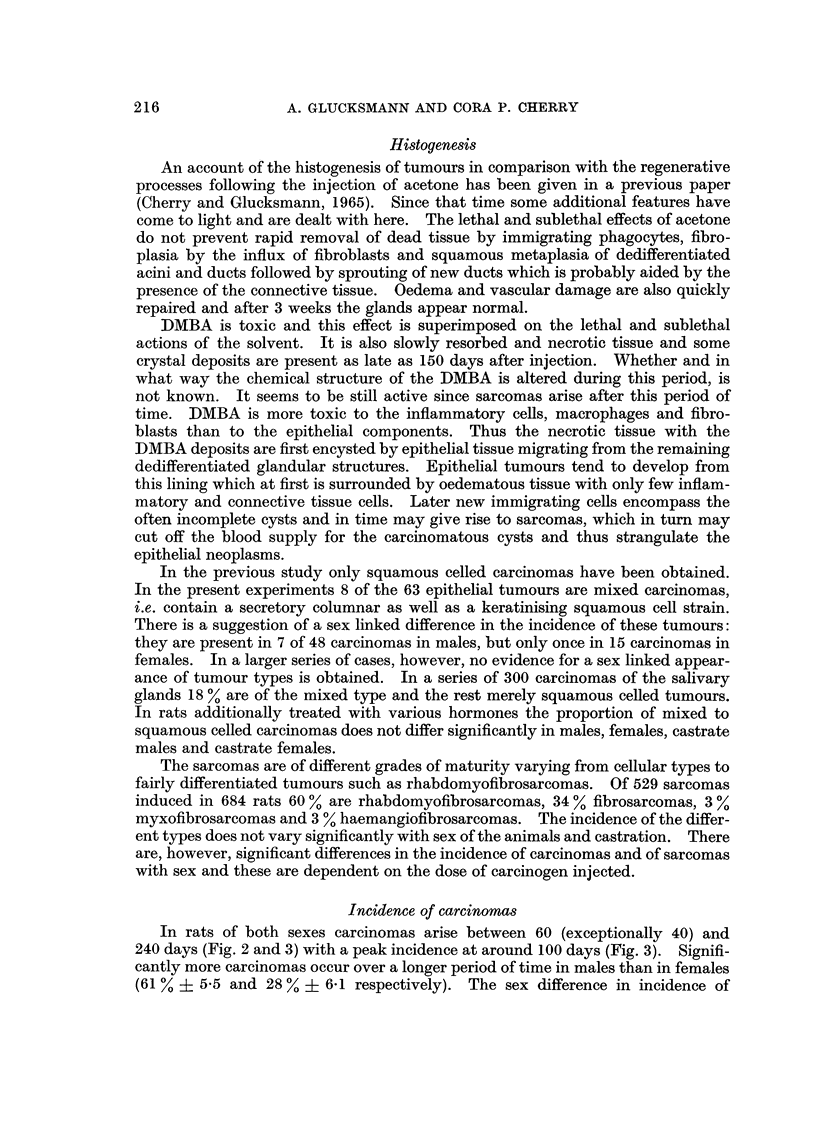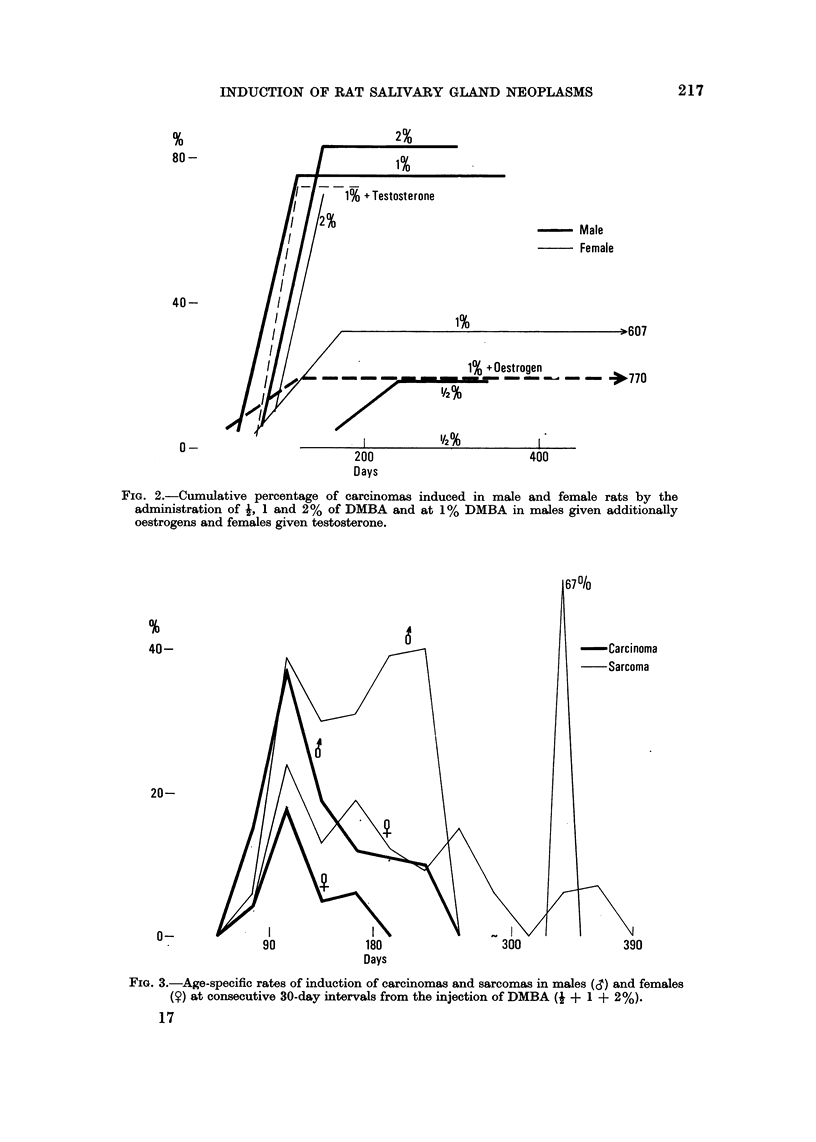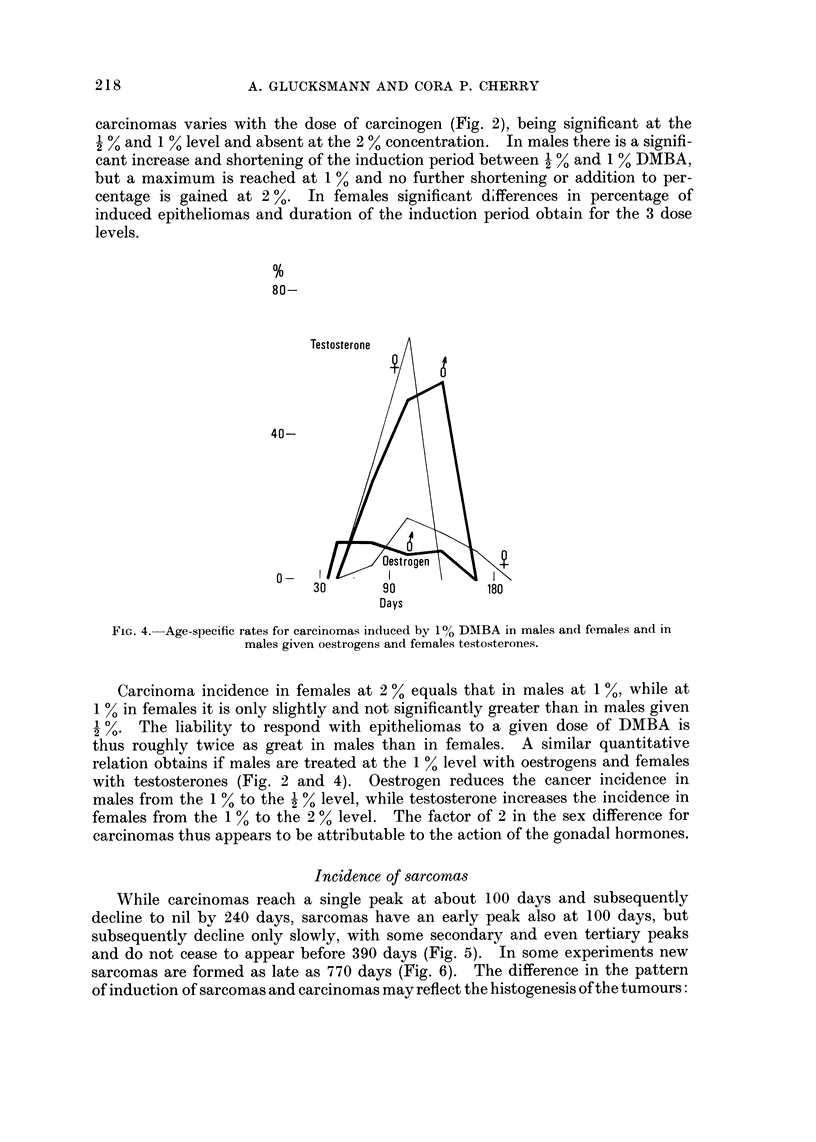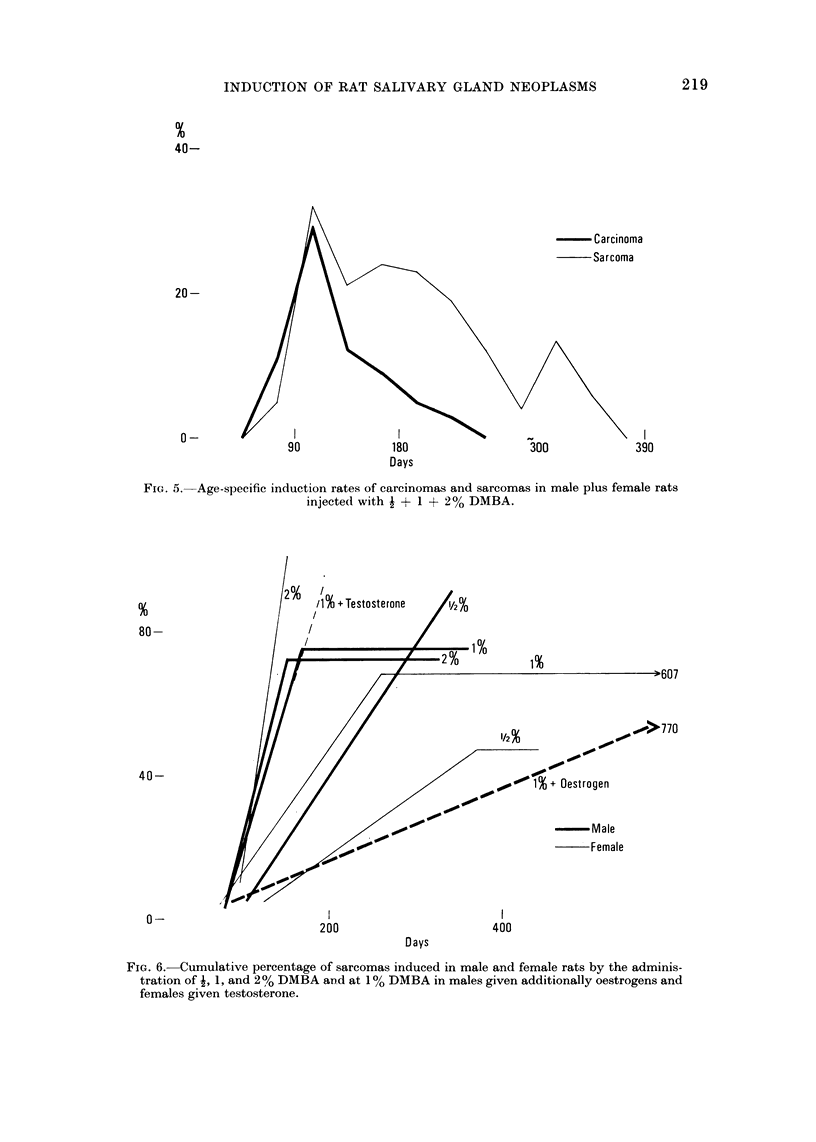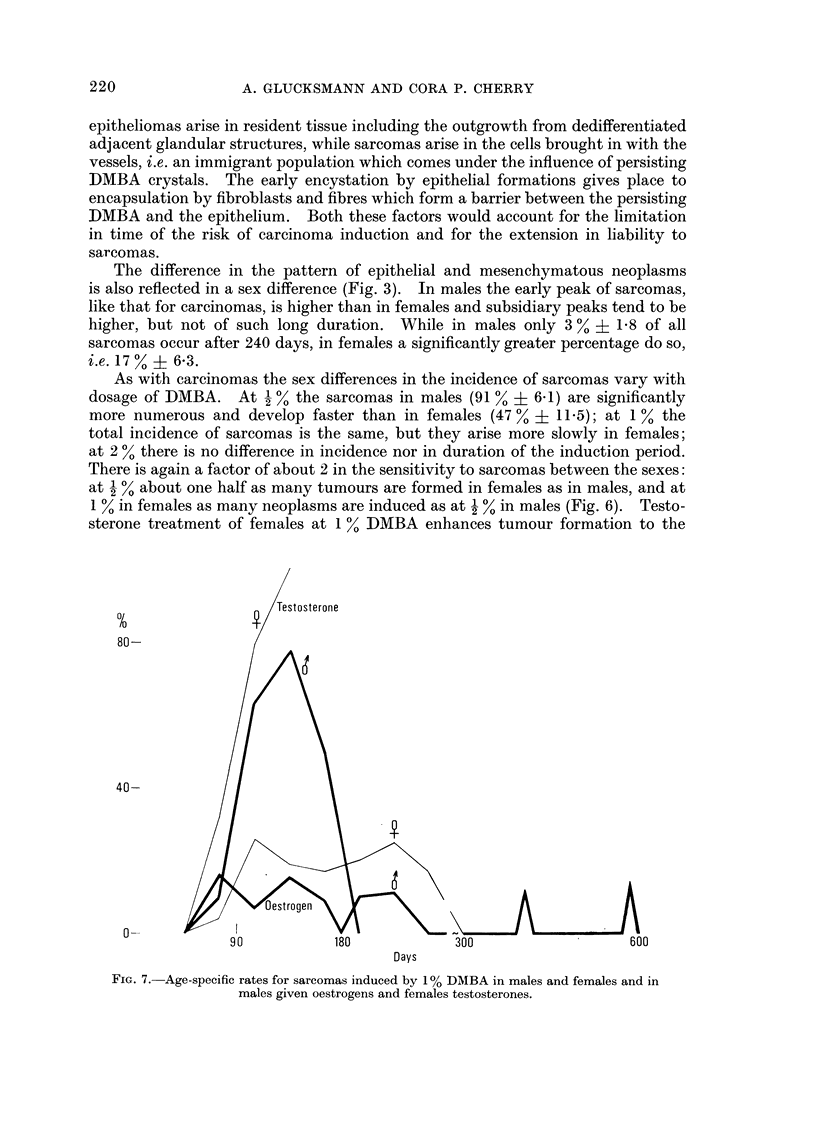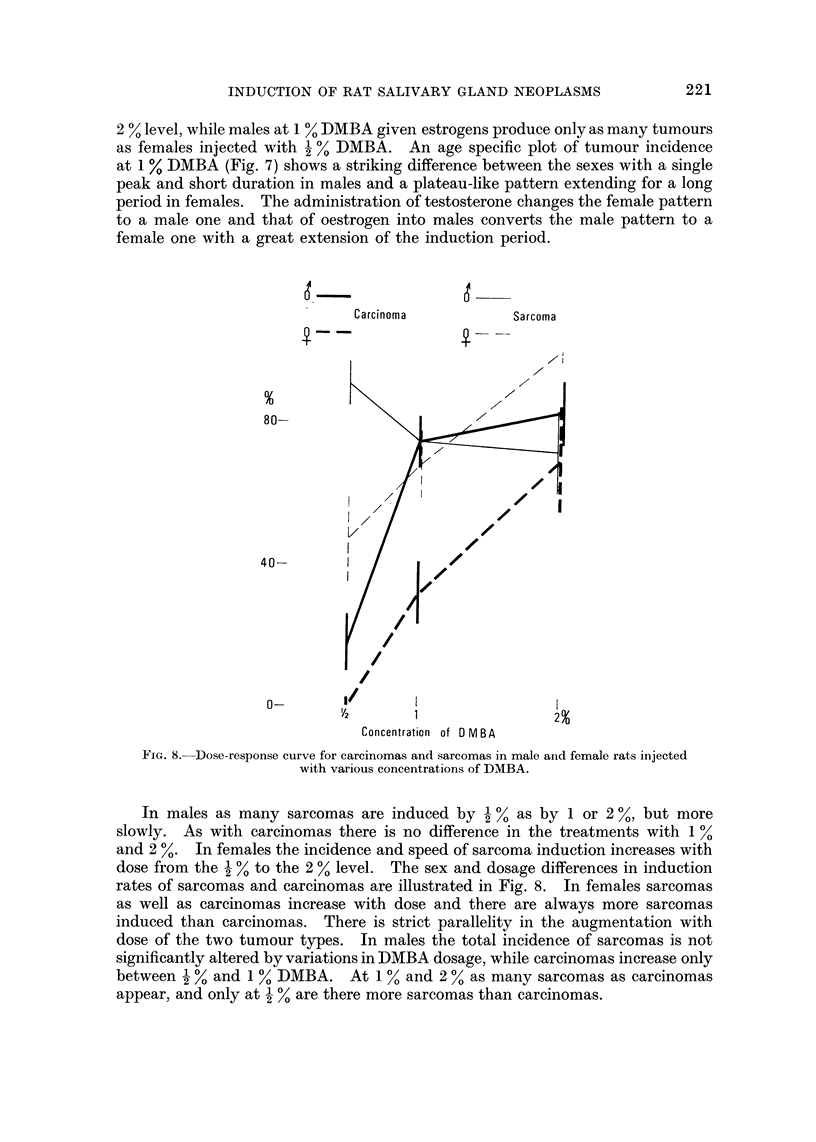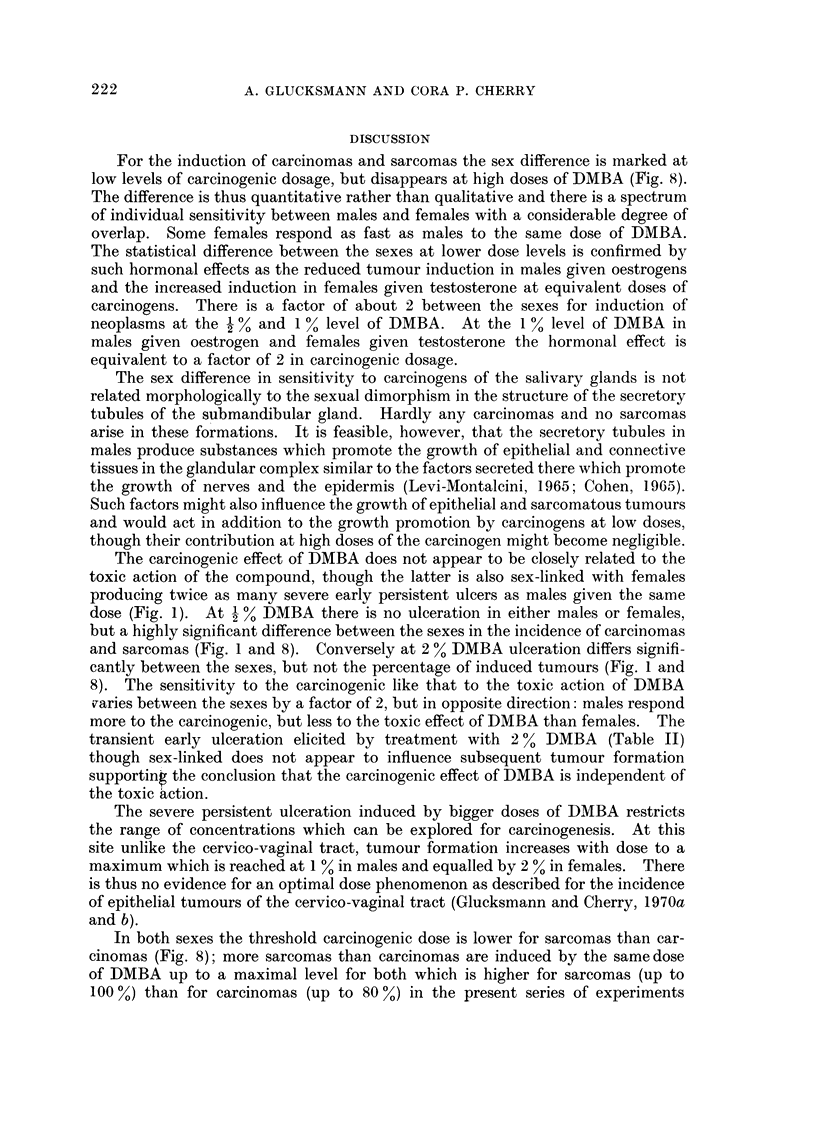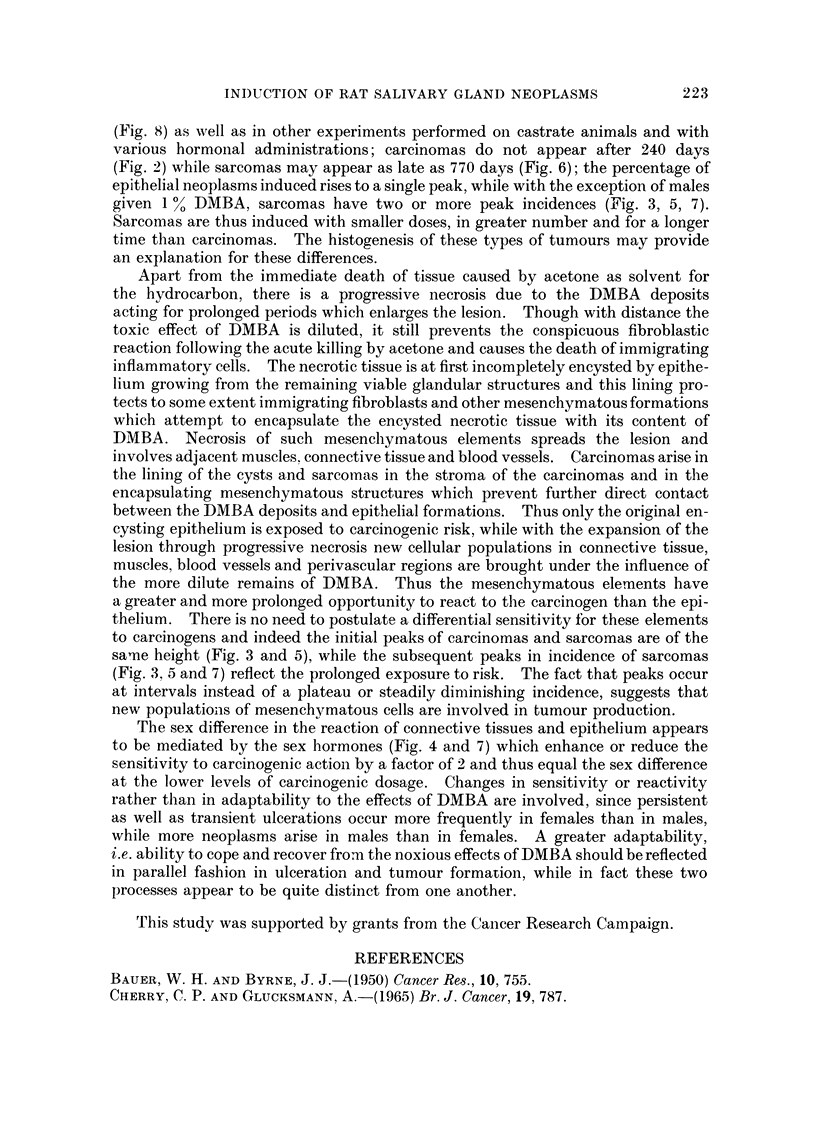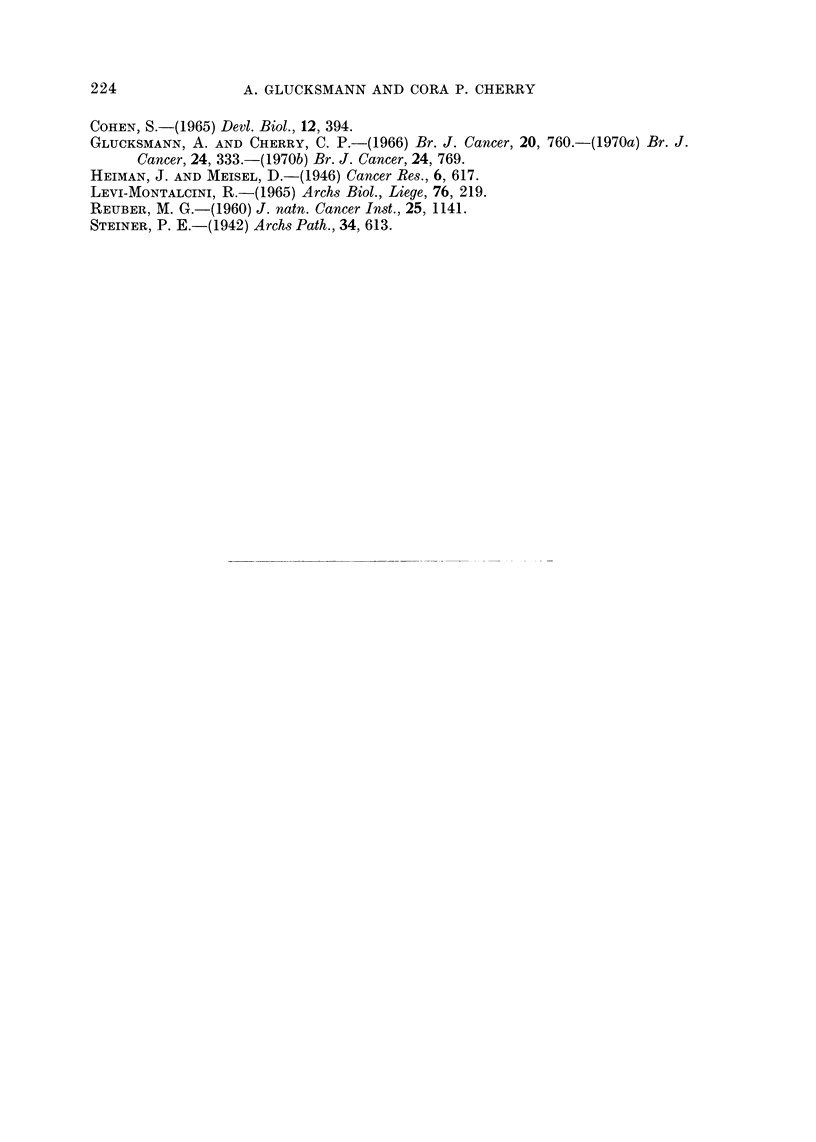# Sex Difference and Carcinogenic Dosage in the Induction of Neoplasms in Salivary Glands of Rats

**DOI:** 10.1038/bjc.1971.28

**Published:** 1971-03

**Authors:** A. Glucksmann, Cora P. Cherry

## Abstract

At low concentrations of DMBA (½% and 1%) twice as many sarcomas and carcinomas of the salivary glands are induced in male as in female rats. Additional oestrogens reduce neoplasms in males by one half while testosterone doubles them in females. The sex difference disappears at the higher dose levels of the carcinogen (2%).

Females are more sensitive than males to the toxic effects of DMBA, though less sensitive to the carcinogenic action.

Carcinomas rise to a single peak within 240 days while sarcomas appear as late as 770 days with secondary and tertiary peaks. This difference in pattern of induction may be due to the formation of a fibrous capsule separating persisting DMBA-deposits from the epithelial structures and thus protecting them from carcinogenic risk.


### SEX DIFFERENCE AND CARCINOGENIC DOSAGE IN THE INDUCTION OF NEOPLASMS IN SALIVARY GLANDS OF RATS

A. GLUCKSMANN AND CORA P. CHERRY

*From the Strangeways Research Laboratory, Cambridge*

Received for publication January 14, 1971

**SUMMARY.—At low concentrations of DMBA (½% and 1%) twice as many sarcomas and carcinomas of the salivary glands are induced in male as in female rats. Additional oestrogens reduce neoplasms in males by one half while testosterone doubles them in females. The sex difference disappears at the higher dose levels of the carcinogen (2%).**

**Females are more sensitive than males to the toxic effects of DMBA, though less sensitive to the carcinogenic action.**

**Carcinomas rise to a single peak within 240 days while sarcomas appear as late as 770 days with secondary and tertiary peaks. This difference in pattern of induction may be due to the formation of a fibrous capsule separating persisting DMBA-deposits from the epithelial structures and thus protecting them from carcinogenic risk.**

THE induction of malignant neoplasms in the salivary glands of rats varies with sex and can be modified by castration and by administration of sex hormones (Glucksmann and Cherry, 1966). For the same dose of the carcinogen tumour incidence is greater in males than in females, and is reduced in males by oestrogens and increased in females by testosterones. At this dose level sarcomas and carcinomas are elicited in equal proportions in males, while in females twice as many sarcomas as carcinomas are induced. The present investigation is concerned with the effect of varying the carcinogenic dosage on the sex difference in tumour induction and the proportion of resulting sarcomas and carcinomas.

Experiments on carcinogenesis in the cervico-vaginal tract of rats have shown that the incidence of sarcomas increases with increasing numbers of weekly applications of the carcinogen, but there is an optimal level of dosage for the induction of epithelial tumours and subsequent inhibition of tumour formation (Glucksmann and Cherry, 1970*a* and *b*). If salivary gland tumours behave similarly, doubling the concentration of the carcinogen might be expected to increase the incidence of sarcomas but to reduce that of carcinomas, while halving the concentration might increase the induction of carcinomas both relatively to sarcomas and absolutely. Dose variation may also affect the sex difference and account for some contradictory findings by different authors. Thus Steiner (1942) and Bauer and Byrne (1950) have not found an influence of sex on carcinogenesis, while Heiman and Meisel (1946), Reuber (1960) and Glucksmann and Cherry (1966) have reported it.

Our investigation shows that the incidence of sarcomas as well as of carcinomas increases with dose of carcinogen and that the sex difference disappears with the highest dose of carcinogen used.

### MATERIALS AND METHODS

Two to three months old male and female hooded rats of the Lister strain, random bred in this laboratory as a closed colony since 1940, were used for the experiments. The rats were housed not more than seven to a cage and given water and food pellets of MRC-diet 86 *ad libitum.*

The carcinogen 9,10-dimethyl-1,2-benzanthracene (DMBA, Light & Co. or Sigma) was dissolved in acetone and the concentration was varied to give a $\frac{1}{2}$% or a 1 % solution or a 2 % suspension. Each rat received under ether anaesthesia an injection of 0·1 ml. of one of the carcinogenic solutions into the right and the left salivary gland complex, in a few experiments after and in most without surgical exposure of the salivary glands. For each complex 0·05 ml. was injected in an anterior direction to deposit the carcinogen in the submandibular and closely applied sublingual glands and the other 0·05 ml. was directed posteriorly into the parotid gland. Control animals received a similar quantity of acetone by the same injection technique.

In one group of females a 30 mg. pellet of pure testosterone propionate (Ciba) was implanted subcutaneously at the same time as the injection of 1 % DMBA was administered. Stilboestrol B.P. was given to one group of males in the drinking water in a concentration of 0·1 mg./1000 ml. thus dosing each rat with about 2 $\mu$g. per day. This treatment was started one day after the injection of 1 % DMBA.

TABLE I.—*Treatment Groups, Sex and Number of Rats at Risk*

| Injection into salivary glands of | Sex | Number at risk |
|---|---|---|
| Acetone | ♂ | 7 |
| Acetone | ♀ | 7 |
| $\frac{1}{2}$% DMBA in acetone | ♂ | 22 |
| $\frac{1}{2}$% DMBA in acetone | ♀ | 19 |
| 1% DMBA in acetone | ♂ | 40 |
| 1% DMBA in acetone | ♀ | 25 |
| 1% DMBA in acetone* | ♂ | 19 |
| 1% DMBA in acetone† | ♀ | 16 |
| 2% DMBA in acetone | ♂ | 18 |
| 2% DMBA in acetone | ♀ | 10 |

* This group received additional treatment with stilboestrol.
† This group received additional treatment with testosterone.

The rats were examined at weekly intervals, swelling or ulceration in the neck noted and, apart from those in which severe, protracted ulceration necessitated early killing, the animals were allowed to survive until clinical signs of tumours in the neck appeared. Only those surviving for more than 60 days were considered at risk. The details of treatment, sex and number of animals in the various experimental groups are given in Table I. Each group consisted initially of 21 to 44 rats.

At autopsy the right and left salivary gland complexes were dissected off the tumours where possible and fixed separately in Bouin's fluid, dehydrated and embedded in paraffin. The blocks were cut serially and every fifth section taken. The tumours were fixed in Zenker-acetic as were the following additional tissues: pituitary, thyroid, thymus, lungs, liver, spleen, kidneys, adrenals, gonads with uteri and cervico-vaginal tract or seminal vesicles and prostate. The material was processed in routine manner, embedded in paraffin and sectioned at 6 or 8 $\mu$ depending on organ; the endocrine glands were sectioned serially.

Sections were stained with haematoxylin-eosin, the periodic acid-Schiff technique (PAS) after diastase digestion, Southgate's mucicarmine, Trevan's alcian blue-basic fuchsin method, Van Gieson or with carmalum-orange G-aniline blue.

*Calculation of results*

For the age-specific induction rates the number of tumour-bearing animals amongst those at risk for consecutive 30-day periods was plotted at the 15-day interval.

### RESULTS

*Early ulceration in relation to dosage of DMBA and sex*

Injection of DMBA in acetone is followed rapidly by a swelling in the neck which enlarges at varying rates. In some rats this expansion leads to breakage of the overlying skin during the second week resulting in a primary ulceration which may be of such severity and persistence as to necessitate the killing of the animals. In others the ulceration is slight, heals by the 40th day or is entirely

FIG. 1.—Percentage of male (♂) and female (♀) rats killed because of severe persistent ulceration early after injection of ½, 1 and 2% of DMBA.

absent. The lump in the neck region enlarges and becomes harder and if composed of malignant tissue may cause a secondary ulceration. In rats which fail to produce tumours, the original swelling is resorbed and the only macroscopic change consists of some fibrosis of the glandular capsules.

In rats injected with acetone only, an initial swelling in the neck region is quickly reduced and resorbed and the injected region appears entirely normal after about 3 weeks. In other experiments olive oil used as solvent produces little swelling, but leaves behind a granulomatous reaction. Neither acetone nor olive oil injected controls have ever produced any lumps leading to early ulceration. Those given DMBA (1 %) in olive oil (Cherry and Glucksmann, 1965) produce swellings and tumours, but have not suffered from early ulceration, though secondary ulcers due to malignancy have occurred.

Early ulceration following injection of DMBA in acetone varies in incidence and severity with concentration of the DMBA and sex of the rats (Fig. 1). No early ulceration follows after treatment with ½ % DMBA in either males or females. After 1 % DMBA, 7 % of 44 males and 15 % of 32 females have been killed within 40 days because of severe and persisting ulcers in the neck. At 2 % DMBA severe ulceration has occurred in 24 % of 25 males and 48 % of 23 females. The difference is not quite significant at the 95 % confidence level. There is, however, a significant difference between the sexes if rats treated at the 1 % and 2 % level are considered: $13 \pm 4$ % of 69 males are afflicted as against $29 \pm 6{\cdot}1$ % of 55 females (Diff: $16 \pm 7{\cdot}3$). As the graph shows, the incidence of early ulcers increases rapidly with concentration of the carcinogen.

At the 2 % level a mild and transient ulceration has been seen in 6 of 18 males and 5 of 10 females. It has started by the 15th day and healed by 40 days in all except 2 males in whom the ulceration has been delayed to about 40 days and the healing to between 68 and 90 days. All 11 animals with ulcers have tumours subsequently and only 2 of 17 are without ulceration and tumours (Table II).

TABLE II.—*Tumour Incidence in Rats Injected with 2 % DMBA in Relation to Transient Early Ulceration*

| Sex | Ulceration | No. | Carcinoma | Carcinoma + sarcoma | Sarcoma | No tumour |
|---|---|---|---|---|---|---|
| ♂ | + | 6 | 2 | 3 | 1 | 0 |
| | − | 12 | 1 | 9 | 0 | 2 |
| | | 18 | 3 | 12 | 1 | 2 |
| ♀ | + | 5 | 0 | 2 | 3 | 0 |
| | − | 5 | 0 | 5 | 0 | 0 |
| | | 10 | 0 | 7 | 3 | 0 |

There is no convincing correlation between type of tumour developing later and early transient ulceration. Most animals have both sarcomas and carcinomas, though three females with ulcers have sarcomas only. In males with prior ulceration there are two with carcinomas only and one with a sarcoma only. These minor differences are more likely to be accounted for by the sex differences in the incidence of tumour types than by the preceding ulceration. At the ½ % level there is no ulceration, but carcinomas develop in males though not in females.

*Histogenesis*

An account of the histogenesis of tumours in comparison with the regenerative processes following the injection of acetone has been given in a previous paper (Cherry and Glucksmann, 1965). Since that time some additional features have come to light and are dealt with here. The lethal and sublethal effects of acetone do not prevent rapid removal of dead tissue by immigrating phagocytes, fibroplasia by the influx of fibroblasts and squamous metaplasia of dedifferentiated acini and ducts followed by sprouting of new ducts which is probably aided by the presence of the connective tissue. Oedema and vascular damage are also quickly repaired and after 3 weeks the glands appear normal.

DMBA is toxic and this effect is superimposed on the lethal and sublethal actions of the solvent. It is also slowly resorbed and necrotic tissue and some crystal deposits are present as late as 150 days after injection. Whether and in what way the chemical structure of the DMBA is altered during this period, is not known. It seems to be still active since sarcomas arise after this period of time. DMBA is more toxic to the inflammatory cells, macrophages and fibroblasts than to the epithelial components. Thus the necrotic tissue with the DMBA deposits are first encysted by epithelial tissue migrating from the remaining dedifferentiated glandular structures. Epithelial tumours tend to develop from this lining which at first is surrounded by oedematous tissue with only few inflammatory and connective tissue cells. Later new immigrating cells encompass the often incomplete cysts and in time may give rise to sarcomas, which in turn may cut off the blood supply for the carcinomatous cysts and thus strangulate the epithelial neoplasms.

In the previous study only squamous celled carcinomas have been obtained. In the present experiments 8 of the 63 epithelial tumours are mixed carcinomas, *i.e.* contain a secretory columnar as well as a keratinising squamous cell strain. There is a suggestion of a sex linked difference in the incidence of these tumours: they are present in 7 of 48 carcinomas in males, but only once in 15 carcinomas in females. In a larger series of cases, however, no evidence for a sex linked appearance of tumour types is obtained. In a series of 300 carcinomas of the salivary glands 18 % are of the mixed type and the rest merely squamous celled tumours. In rats additionally treated with various hormones the proportion of mixed to squamous celled carcinomas does not differ significantly in males, females, castrate males and castrate females.

The sarcomas are of different grades of maturity varying from cellular types to fairly differentiated tumours such as rhabdomyofibrosarcomas. Of 529 sarcomas induced in 684 rats 60 % are rhabdomyofibrosarcomas, 34 % fibrosarcomas, 3 % myxofibrosarcomas and 3 % haemangiofibrosarcomas. The incidence of the different types does not vary significantly with sex of the animals and castration. There are, however, significant differences in the incidence of carcinomas and of sarcomas with sex and these are dependent on the dose of carcinogen injected.

*Incidence of carcinomas*

In rats of both sexes carcinomas arise between 60 (exceptionally 40) and 240 days (Fig. 2 and 3) with a peak incidence at around 100 days (Fig. 3). Significantly more carcinomas occur over a longer period of time in males than in females (61 % $\pm$ 5·5 and 28 % $\pm$ 6·1 respectively). The sex difference in incidence of

FIG. 2.—Cumulative percentage of carcinomas induced in male and female rats by the administration of ½, 1 and 2% of DMBA and at 1% DMBA in males given additionally oestrogens and females given testosterone.

FIG. 3.—Age-specific rates of induction of carcinomas and sarcomas in males (♂) and females (♀) at consecutive 30-day intervals from the injection of DMBA (½ + 1 + 2%).

carcinomas varies with the dose of carcinogen (Fig. 2), being significant at the ½ % and 1 % level and absent at the 2 % concentration. In males there is a significant increase and shortening of the induction period between ½ % and 1 % DMBA, but a maximum is reached at 1 % and no further shortening or addition to percentage is gained at 2 %. In females significant differences in percentage of induced epitheliomas and duration of the induction period obtain for the 3 dose levels.

Fig. 4.—Age-specific rates for carcinomas induced by 1 % DMBA in males and females and in males given oestrogens and females testosterones.

Carcinoma incidence in females at 2 % equals that in males at 1 %, while at 1 % in females it is only slightly and not significantly greater than in males given ½ %. The liability to respond with epitheliomas to a given dose of DMBA is thus roughly twice as great in males than in females. A similar quantitative relation obtains if males are treated at the 1 % level with oestrogens and females with testosterones (Fig. 2 and 4). Oestrogen reduces the cancer incidence in males from the 1 % to the ½ % level, while testosterone increases the incidence in females from the 1 % to the 2 % level. The factor of 2 in the sex difference for carcinomas thus appears to be attributable to the action of the gonadal hormones.

*Incidence of sarcomas*

While carcinomas reach a single peak at about 100 days and subsequently decline to nil by 240 days, sarcomas have an early peak also at 100 days, but subsequently decline only slowly, with some secondary and even tertiary peaks and do not cease to appear before 390 days (Fig. 5). In some experiments new sarcomas are formed as late as 770 days (Fig. 6). The difference in the pattern of induction of sarcomas and carcinomas may reflect the histogenesis of the tumours:

FIG. 5.—Age-specific induction rates of carcinomas and sarcomas in male plus female rats injected with ½ + 1 + 2% DMBA.

FIG. 6.—Cumulative percentage of sarcomas induced in male and female rats by the administration of ½, 1, and 2% DMBA and at 1% DMBA in males given additionally oestrogens and females given testosterone.

epitheliomas arise in resident tissue including the outgrowth from dedifferentiated adjacent glandular structures, while sarcomas arise in the cells brought in with the vessels, *i.e.* an immigrant population which comes under the influence of persisting DMBA crystals. The early encystation by epithelial formations gives place to encapsulation by fibroblasts and fibres which form a barrier between the persisting DMBA and the epithelium. Both these factors would account for the limitation in time of the risk of carcinoma induction and for the extension in liability to sarcomas.

The difference in the pattern of epithelial and mesenchymatous neoplasms is also reflected in a sex difference (Fig. 3). In males the early peak of sarcomas, like that for carcinomas, is higher than in females and subsidiary peaks tend to be higher, but not of such long duration. While in males only 3 % ± 1·8 of all sarcomas occur after 240 days, in females a significantly greater percentage do so, *i.e.* 17 % ± 6·3.

As with carcinomas the sex differences in the incidence of sarcomas vary with dosage of DMBA. At ½ % the sarcomas in males (91 % ± 6·1) are significantly more numerous and develop faster than in females (47 % ± 11·5); at 1 % the total incidence of sarcomas is the same, but they arise more slowly in females; at 2 % there is no difference in incidence nor in duration of the induction period. There is again a factor of about 2 in the sensitivity to sarcomas between the sexes: at ½ % about one half as many tumours are formed in females as in males, and at 1 % in females as many neoplasms are induced as at ½ % in males (Fig. 6). Testosterone treatment of females at 1 % DMBA enhances tumour formation to the

FIG. 7.—Age-specific rates for sarcomas induced by 1% DMBA in males and females and in males given oestrogens and females testosterones.

2 % level, while males at 1 % DMBA given estrogens produce only as many tumours as females injected with ½ % DMBA. An age specific plot of tumour incidence at 1 % DMBA (Fig. 7) shows a striking difference between the sexes with a single peak and short duration in males and a plateau-like pattern extending for a long period in females. The administration of testosterone changes the female pattern to a male one and that of oestrogen into males converts the male pattern to a female one with a great extension of the induction period.

FIG. 8.—Dose-response curve for carcinomas and sarcomas in male and female rats injected with various concentrations of DMBA.

In males as many sarcomas are induced by ½ % as by 1 or 2 %, but more slowly. As with carcinomas there is no difference in the treatments with 1 % and 2 %. In females the incidence and speed of sarcoma induction increases with dose from the ½ % to the 2 % level. The sex and dosage differences in induction rates of sarcomas and carcinomas are illustrated in Fig. 8. In females sarcomas as well as carcinomas increase with dose and there are always more sarcomas induced than carcinomas. There is strict parallelity in the augmentation with dose of the two tumour types. In males the total incidence of sarcomas is not significantly altered by variations in DMBA dosage, while carcinomas increase only between ½ % and 1 % DMBA. At 1 % and 2 % as many sarcomas as carcinomas appear, and only at ½ % are there more sarcomas than carcinomas.

### DISCUSSION

For the induction of carcinomas and sarcomas the sex difference is marked at low levels of carcinogenic dosage, but disappears at high doses of DMBA (Fig. 8). The difference is thus quantitative rather than qualitative and there is a spectrum of individual sensitivity between males and females with a considerable degree of overlap. Some females respond as fast as males to the same dose of DMBA. The statistical difference between the sexes at lower dose levels is confirmed by such hormonal effects as the reduced tumour induction in males given oestrogens and the increased induction in females given testosterone at equivalent doses of carcinogens. There is a factor of about 2 between the sexes for induction of neoplasms at the $\frac{1}{2}$% and 1% level of DMBA. At the 1% level of DMBA in males given oestrogen and females given testosterone the hormonal effect is equivalent to a factor of 2 in carcinogenic dosage.

The sex difference in sensitivity to carcinogens of the salivary glands is not related morphologically to the sexual dimorphism in the structure of the secretory tubules of the submandibular gland. Hardly any carcinomas and no sarcomas arise in these formations. It is feasible, however, that the secretory tubules in males produce substances which promote the growth of epithelial and connective tissues in the glandular complex similar to the factors secreted there which promote the growth of nerves and the epidermis (Levi-Montalcini, 1965; Cohen, 1965). Such factors might also influence the growth of epithelial and sarcomatous tumours and would act in addition to the growth promotion by carcinogens at low doses, though their contribution at high doses of the carcinogen might become negligible.

The carcinogenic effect of DMBA does not appear to be closely related to the toxic action of the compound, though the latter is also sex-linked with females producing twice as many severe early persistent ulcers as males given the same dose (Fig. 1). At $\frac{1}{2}$% DMBA there is no ulceration in either males or females, but a highly significant difference between the sexes in the incidence of carcinomas and sarcomas (Fig. 1 and 8). Conversely at 2% DMBA ulceration differs significantly between the sexes, but not the percentage of induced tumours (Fig. 1 and 8). The sensitivity to the carcinogenic like that to the toxic action of DMBA varies between the sexes by a factor of 2, but in opposite direction: males respond more to the carcinogenic, but less to the toxic effect of DMBA than females. The transient early ulceration elicited by treatment with 2% DMBA (Table II) though sex-linked does not appear to influence subsequent tumour formation supporting the conclusion that the carcinogenic effect of DMBA is independent of the toxic action.

The severe persistent ulceration induced by bigger doses of DMBA restricts the range of concentrations which can be explored for carcinogenesis. At this site unlike the cervico-vaginal tract, tumour formation increases with dose to a maximum which is reached at 1% in males and equalled by 2% in females. There is thus no evidence for an optimal dose phenomenon as described for the incidence of epithelial tumours of the cervico-vaginal tract (Glucksmann and Cherry, 1970*a* and *b*).

In both sexes the threshold carcinogenic dose is lower for sarcomas than carcinomas (Fig. 8); more sarcomas than carcinomas are induced by the same dose of DMBA up to a maximal level for both which is higher for sarcomas (up to 100%) than for carcinomas (up to 80%) in the present series of experiments

(Fig. 8) as well as in other experiments performed on castrate animals and with various hormonal administrations; carcinomas do not appear after 240 days (Fig. 2) while sarcomas may appear as late as 770 days (Fig. 6); the percentage of epithelial neoplasms induced rises to a single peak, while with the exception of males given 1% DMBA, sarcomas have two or more peak incidences (Fig. 3, 5, 7). Sarcomas are thus induced with smaller doses, in greater number and for a longer time than carcinomas. The histogenesis of these types of tumours may provide an explanation for these differences.

Apart from the immediate death of tissue caused by acetone as solvent for the hydrocarbon, there is a progressive necrosis due to the DMBA deposits acting for prolonged periods which enlarges the lesion. Though with distance the toxic effect of DMBA is diluted, it still prevents the conspicuous fibroblastic reaction following the acute killing by acetone and causes the death of immigrating inflammatory cells. The necrotic tissue is at first incompletely encysted by epithelium growing from the remaining viable glandular structures and this lining protects to some extent immigrating fibroblasts and other mesenchymatous formations which attempt to encapsulate the encysted necrotic tissue with its content of DMBA. Necrosis of such mesenchymatous elements spreads the lesion and involves adjacent muscles, connective tissue and blood vessels. Carcinomas arise in the lining of the cysts and sarcomas in the stroma of the carcinomas and in the encapsulating mesenchymatous structures which prevent further direct contact between the DMBA deposits and epithelial formations. Thus only the original encysting epithelium is exposed to carcinogenic risk, while with the expansion of the lesion through progressive necrosis new cellular populations in connective tissue, muscles, blood vessels and perivascular regions are brought under the influence of the more dilute remains of DMBA. Thus the mesenchymatous elements have a greater and more prolonged opportunity to react to the carcinogen than the epithelium. There is no need to postulate a differential sensitivity for these elements to carcinogens and indeed the initial peaks of carcinomas and sarcomas are of the same height (Fig. 3 and 5), while the subsequent peaks in incidence of sarcomas (Fig. 3, 5 and 7) reflect the prolonged exposure to risk. The fact that peaks occur at intervals instead of a plateau or steadily diminishing incidence, suggests that new populations of mesenchymatous cells are involved in tumour production.

The sex difference in the reaction of connective tissues and epithelium appears to be mediated by the sex hormones (Fig. 4 and 7) which enhance or reduce the sensitivity to carcinogenic action by a factor of 2 and thus equal the sex difference at the lower levels of carcinogenic dosage. Changes in sensitivity or reactivity rather than in adaptability to the effects of DMBA are involved, since persistent as well as transient ulcerations occur more frequently in females than in males, while more neoplasms arise in males than in females. A greater adaptability, *i.e.* ability to cope and recover from the noxious effects of DMBA should be reflected in parallel fashion in ulceration and tumour formation, while in fact these two processes appear to be quite distinct from one another.

This study was supported by grants from the Cancer Research Campaign.

### REFERENCES

BAUER, W. H. AND BYRNE, J. J.—(1950) *Cancer Res.*, **10**, 755.
CHERRY, C. P. AND GLUCKSMANN, A.—(1965) *Br. J. Cancer*, **19**, 787.

COHEN, S.—(1965) *Devl. Biol.*, **12**, 394.
GLUCKSMANN, A. AND CHERRY, C. P.—(1966) *Br. J. Cancer*, **20**, 760.—(1970*a*) *Br. J. Cancer*, **24**, 333.—(1970*b*) *Br. J. Cancer*, **24**, 769.
HEIMAN, J. AND MEISEL, D.—(1946) *Cancer Res.*, **6**, 617.
LEVI-MONTALCINI, R.—(1965) *Archs Biol., Liege*, **76**, 219.
REUBER, M. G.—(1960) *J. natn. Cancer Inst.*, **25**, 1141.
STEINER, P. E.—(1942) *Archs Path.*, **34**, 613.